# Hypothalamic circuits regulating appetite and energy homeostasis: pathways to obesity

**DOI:** 10.1242/dmm.026609

**Published:** 2017-06-01

**Authors:** Katharina Timper, Jens C. Brüning

**Affiliations:** 1 Max Planck Institute for Metabolism Research, Department of Neuronal Control of Metabolism, Gleueler Str. 50, Cologne 50931, Germany; 2 Center for Endocrinology, Diabetes and Preventive Medicine (CEDP), University Hospital Cologne, Kerpener Str. 26, Cologne 50924, Germany; 3 Excellence Cluster on Cellular Stress Responses in Aging Associated Diseases (CECAD) and Center of Molecular Medicine Cologne (CMMC), University of Cologne, Joseph-Stelzmann-Str. 26, Cologne 50931, Germany; 4 National Center for Diabetes Research (DZD), Ingolstädter Land Str. 1, Neuherberg 85764, Germany

**Keywords:** Obesity, Type 2 diabetes mellitus, Glucose homeostasis, Hypothalamus, CNS

## Abstract

The ‘obesity epidemic’ represents a major global socioeconomic burden that urgently calls for a better understanding of the underlying causes of increased weight gain and its associated metabolic comorbidities, such as type 2 diabetes mellitus and cardiovascular diseases. Improving our understanding of the cellular basis of obesity could set the stage for the development of new therapeutic strategies. The CNS plays a pivotal role in the regulation of energy and glucose homeostasis. Distinct neuronal cell populations, particularly within the arcuate nucleus of the hypothalamus, sense the nutrient status of the organism and integrate signals from peripheral hormones including pancreas-derived insulin and adipocyte-derived leptin to regulate calorie intake, glucose metabolism and energy expenditure. The arcuate neurons are tightly connected to other specialized neuronal subpopulations within the hypothalamus, but also to various extrahypothalamic brain regions, allowing a coordinated behavioral response. This At a Glance article gives an overview of the recent knowledge, mainly derived from rodent models, regarding the CNS-dependent regulation of energy and glucose homeostasis, and illustrates how dysregulation of the neuronal networks involved can lead to overnutrition and obesity. The potential impact of recent research findings in the field on therapeutic treatment strategies for human obesity is also discussed.

## Abstract (precis)

**Summary:** This at a glance article gives an overview of the recent knowledge mainly derived from rodent models regarding the CNS-dependent regulation of energy and glucose homeostasis, and depicts how dysregulation of the involved neuronal networks promotes overnutrition and obesity.

## Introduction

The number of overweight and obese people worldwide has increased over recent years, giving rise to a global obesity epidemic. According to the World Health Organization (WHO), in 2014, more than 1.9 billion adults were overweight, of which over 600 million were classified as clinically obese (WHO, 2015). Perhaps even more alarmingly, 42 million children under the age of 5 were overweight or obese in 2013 (WHO, 2015). Obesity is a major risk factor for associated comorbidities such as cardiovascular diseases (Kim et al., 2015), type 2 diabetes mellitus, cancer (Calle et al., 2003) and musculoskeletal disorders, and is associated with an increased overall mortality relative to non-obese individuals (Adams et al., 2006).

**Figure:**
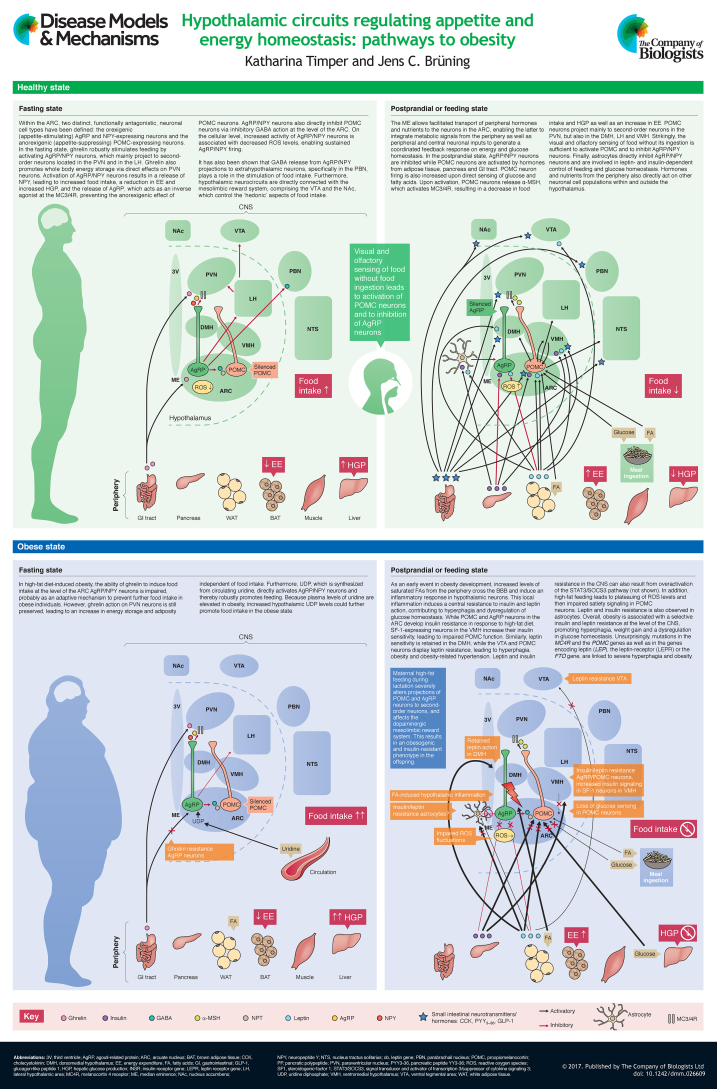
A high-resolution version of the poster is available for downloading at http://dmm.biologists.org/lookup/doi/10.1242/dmm.026609.supplemental.

Obesity results from the dysregulation of energy metabolism (Crowley et al., 2002). The central nervous system (CNS) plays a key role in sensing and controlling the energy status of the organism (Myers and Olson, 2012), and the hypothalamus in particular has emerged as an integrating, superordinate master regulator of whole-body energy homeostasis. Obesity has long been considered to be the result of a lack of discipline and effort to reduce calorie intake and to increase physical activity, which has led to a fundamental and still present social weight-related stigmatization of affected individuals (Friedman, 2004). However, extensive research in both humans and various murine model systems over recent decades has revealed that a complex interplay of genes and environmental factors that impact CNS control of food intake and energy homeostasis pave the way for the development of obesity. In this review and its accompanying poster, we provide a snapshot of the hypothalamic neurocircuits that regulate the homeostatic control of energy metabolism and feeding. We also give an overview of the pleiotropic factors involved in the regulation and dysregulation of the homeostatic hypothalamic system in the context of obesity, including hypothalamic inflammation as well as insulin and leptin resistance. Later, we discuss the impact of a maternal high-fat diet and obesity on offspring and highlight the importance of mitochondrial dynamics and genetic factors involved in the development of obesity at the level of the hypothalamic neurocircuits. Thereby, most of the molecular insights summarized in this article are derived from studies in rodent model organisms. Finally, we provide our outlook on recent perspectives on CNS-dependent control of feeding behavior and metabolism, and future therapeutic approaches to tackle dysfunctional regulation of central energy homeostasis.

## Hypothalamic neuronal circuits controlling feeding behavior and energy homeostasis

### Key hypothalamic nuclei

The hypothalamus is one of the best-studied and most important brain regions involved in the central control of feeding and energy expenditure. In particular, the arcuate nucleus (ARC) within the hypothalamus is critical for the regulation of feeding and metabolism (Myers and Olson, 2012). The ARC is located near to the median eminence (ME; see poster), a circumventricular organ that is rich in fenestrated capillaries that lead to a ‘leaky’ blood-brain barrier (BBB). The ME facilitates transport of peripheral hormonal and nutrient signals and their sensing by the ARC neurons (Rodríguez et al., 2010). Thereby, the ARC integrates hormonal and nutritional metabolic signals from the peripheral circulation as well as peripheral and central neuronal inputs to generate a coordinated feedback response.

There are two distinct, functionally antagonistic types of neurons in the ARC: the orexigenic (appetite-stimulating) neuropeptide Y (NPY) and agouti-related peptide (AgRP)-expressing AgRP/NPY neurons and the anorexigenic (appetite-suppressing) pro-opiomelanocortin (POMC)-expressing POMC neurons (see poster) (Gropp et al., 2005; Balthasar et al., 2005). POMC neurons project mainly to second-order neurons in the paraventricular hypothalamic nucleus (PVN), but also to the dorsomedial hypothalamus (DMH), the lateral hypothalamus (LH) and the ventromedial hypothalamus (VMH) (see poster) (Kleinridders et al., 2009; Waterson and Horvath, 2015). These second-order neurons further process the received information and project to multiple neurocircuits outside of the hypothalamus (extrahypothalamic), leading to an integrated response on energy intake and expenditure, respectively (Roh et al., 2016). Neurons within the PVN control sympathetic outflow to peripheral organs (Kannan et al., 1989) and secrete a variety of regulatory neuropeptides (Roh et al., 2016). Destruction of the PVN leads to overeating and obesity (Leibowitz et al., 1981), pointing to the important role of PVN neurons for the inhibitory control of food intake. Moreover, destruction of the VMH results in hyperphagia and obesity (Shimizu et al., 1987), whereas deletion of the DMH (Bellinger and Bernardis, 2002) and the LH (Milam et al., 1980) produces a hypophagic, lean phenotype.

Upon nutrient ingestion, POMC is cleaved to α-melanocyte-stimulating hormone (α-MSH) which is released from POMC-axons to activate melanocortin 3 and 4 receptors (MC3/4R) on downstream neurons, including neurons in the PVN (see poster; Healthy postprandial or feeding state), resulting in a decrease in food intake and an increase in energy expenditure (Könner et al., 2009). Although MC4R expression is widely distributed among different brain regions (Gantz et al., 1993a,b; Liu et al., 2003), within the hypothalamus, the PVN displays the highest MC4R expression and is considered to host the predominant energy-intake-regulating MC4R population within the CNS (see Tao, 2010; Krashes et al., 2016 for reviews). Consistent with this, studies in mice have shown that disruption of the MC4R, and specifically in the PVN, results in obesity as a result of hyperphagia and reduced energy expenditure, along with deteriorations in glucose homeostasis (Huszar et al., 1997; Balthasar et al., 2005). Downstream mediators likely to be involved in transducing the effects of MC4R activation on food intake regulation are brain-derived neurotrophic factor (BDNF) (Xu et al., 2003; Nicholson et al., 2007), corticotropin-releasing hormone (CRH) (Lu et al., 2003) and thyrotropin-releasing hormone (TRH) (Fekete et al., 2000; Kim et al., 2000). While food intake is believed to be inhibited via hypothalamic melanocortinergic neurons in a constant manner (Fan et al., 1997), energy expenditure is increased upon MC4R activation due to an increased activity of the sympathetic nervous system leading to brown adipose tissue (BAT) activation (Ste Marie et al., 2000; Voss-Andreae et al., 2007).

Fasting, on the other hand, induces the activation of AgRP/NPY neurons that project also to the PVN and the LH (see poster; Healthy fasting state) (Betley et al., 2013). AgRP/NPY neurons co-release NPY and AgRP. NPY directly stimulates food intake (Stanley and Leibowitz, 1984; Clark et al., 1984) via activation of NPY Y1 (Yokosuka et al., 1999) and Y5 (Parker et al., 2000; Cabrele et al., 2000; McCrea et al., 2000) receptors. Furthermore, NPY reduces energy expenditure via a Y1-receptor-mediated reduction in tyrosine hydroxylase expression in the PVN and the brainstem, which leads to a decreased sympathetic output to the BAT and concomitantly reduced BAT activity (Shi et al., 2013). AgRP acts as an inverse agonist of MC3/4R, thereby preventing the anorexigenic effect of α-MSH on second-order neurons (Ollmann et al., 1997). Furthermore, AgRP/NPY neurons directly inhibit POMC neurons via inhibitory γ-aminobutyric acid (GABA) action at the level of the ARC (Cowley et al., 2001). Interestingly, while optogenetic activation of AgRP→POMC synaptic connections strongly inhibited POMC neuron activity, suppression of POMC neuron activity by AgRP neurons was shown not to be required for acute feeding induction (Atasoy et al., 2012). However, exclusive activation of the ARC→PVN connection strongly induces feeding (Atasoy et al., 2012). GABA release from AgRP/NPY projections to extrahypothalamic neurons, specifically in the parabrachial nucleus (PBN), also plays a role in the stimulation of food intake (Wu et al., 2009, 2012), although the exclusive activation of the AgRP/NPY→PBN interconnection is not sufficient to induce feeding (Atasoy et al., 2012). Recently, a study involving optogenetic activation of AgRP neurons demonstrated that distinct AgRP neuron subpopulations project to different brain regions and that activation of some projections – including those to the PVN and LH, but not to the PBN – are able to independently induce feeding similar to somatic activation of AgRP neurons (Betley et al., 2013).

Of note, POMC and AgRP/NPY neurons also receive activating glutamatergic feedback input from other hypothalamic nuclei such as the VMH and the PVN, whereas PVN neurons receive inhibitory innervation from LH neurons that promote feeding, resulting in a finely tuned response regulating food intake (Waterson and Horvath, 2015). Strikingly, it has very recently been demonstrated that the visual and olfactory sensing of food without ingestion of any nutrients is sufficient to reverse the effects of fasting on POMC and AgRP/NPY neurons (Chen et al., 2015b).

As mentioned above, intrahypothalamic projections from ARC neurons to the PVN not only play a role in the regulation of food intake, but also in energy expenditure: POMC neurons increase (Enriori et al., 2011) while AgRP/NPY neurons decrease (Shi et al., 2013) metabolic activity in BAT (for reviews, see Waterson and Horvath, 2015; Morrison et al., 2014). Thus, in the fasting state, energy expenditure is decreased, and following food intake, energy expenditure is increased. Of note, we recently demonstrated that AgRP/NPY neuron activation regulates BAT glucose uptake through specific projections to the bed nucleus of the stria terminalis (BNST) (Steculorum et al., 2016), a forebrain structure involved in the control of neuroendocrine and autonomic responses, as well as feeding and reward behavior (Dong and Swanson, 2006). Thereby, this structure interconnects key nuclei of the limbic system with hypothalamic and brainstem structures in both rodents and humans (for reviews, see Crestani et al., 2013; Avery et al., 2016).

### Extrahypothalamic neuronal circuits

Hypothalamic nuclei also project to and receive input from other extrahypothalamic brain regions such as the nucleus of the solitary tract (NTS) to regulate food intake and energy expenditure (for reviews, see Sohn et al., 2013a; Schneeberger et al., 2014; Morton et al., 2014; Waterson and Horvath, 2015; Roh et al., 2016; Roh and Kim, 2016). Furthermore, hypothalamic neurocircuits are directly connected to the mesolimbic reward system, comprising the ventral tegmental area (VTA) and the nucleus accumbens (NAc), which control the ‘hedonic’ aspects of food intake. Decreased glucose levels, such as those that occur in the fasting state, activate glutamate and orexin co-expressing neurons in the LH, which project to and excite dopaminergic neurons in the VTA (Sheng et al., 2014). In addition, activation of inhibitory GABAergic LH neurons that project to the VTA (see poster, Fasting state) leads to increased food intake and specifically consummatory behavior, which refers to the drive to receive a caloric reward (Jennings et al., 2015). In line with this, two recent studies showed that activation of GABAergic projection from the LH to the VTA enhances compulsive ‘sucrose seeking’ and therefore feeding-related behavior (Nieh et al., 2015; Barbano et al., 2016). Thus, homeostatic inputs from the hypothalamus are integrated with hedonic feeding signals from mesolimbic pathways and signals from superordinate decision-making centers such as the amygdala, the hippocampus and the prefrontal cortex to generate an orchestrated response on feeding, glucose metabolism and energy homeostasis regulation. Although extensive research in rodent models in recent years has made a vast contribution to deciphering the underlying mechanisms of food-related reward stimuli and decision making (Stuber and Wise, 2016; de Macedo et al., 2016), the interplay of the different components of the reward system contributing to food-related reward in humans are far more complex. Indeed, a number of mostly fMRI-based studies have shed light on the complexity of feeding-related reward mechanisms and brain structures involved in humans (Kringelbach, 2005; Farr et al., 2016; Alonso-Alonso et al., 2015).

In summary, the hypothalamus plays a key role in the regulation of appetite and food intake both in humans and rodents (Yeo and Heisler, 2012). Furthermore, brain regions such as the amygdala and the striatum as well as the VTA, with its dopaminergic projections, are involved in both human and rodent food-related reward (Berridge and Kringelbach, 2008). However, distinct from rodents, in humans, reward and behavioral drives are strongly impacted and directed by cognition (Alonso-Alonso et al., 2015).

### Leptin and insulin as modulators of central control of feeding, glucose and energy homeostasis

Both POMC and AgRP/NPY neurons express receptors for peripheral metabolic hormones such as insulin and leptin. Insulin is secreted from pancreatic β-cells upon nutrient ingestion (Prentki et al., 2013) and plays an important role in the peripheral regulation of energy and glucose homeostasis. It specifically controls peripheral glucose metabolism by suppressing hepatic glucose production (HGP) via direct action on hepatic insulin receptors. However, insulin is also a major factor in the regulation of glucose and energy homeostasis at the level of the hypothalamus (see poster, Healthy feeding state) (Belgardt and Brüning, 2010). Activation of insulin receptors on POMC neurons causes membrane hyperpolarization and reduced firing of these neurons (Könner et al., 2007; Williams et al., 2010) while increasing *POMC* mRNA expression (Benoit et al., 2002). However, a recent publication demonstrated that purified insulin actually excites POMC neurons (Qiu et al., 2014). Interestingly, disruption of insulin signaling from POMC neurons did not affect energy or glucose homeostasis (Könner et al., 2007). However, deletion of both insulin and leptin receptors from POMC neurons deteriorates glucose homeostasis and specifically leads to systemic insulin resistance and impaired fertility in mice (Hill et al., 2010). Concomitant insulin and leptin action on POMC neurons also increases the browning of white fat (Dodd et al., 2015), a process that favors enhanced metabolic activity. Furthermore, a very recent study has shown that insulin action on POMC neurons controls adipose-tissue lipolysis and prevents high-fat-diet-induced liver steatosis (Shin et al., 2017). On AgRP/NPY neurons, insulin action is required for the suppressive effect of insulin on HGP. Insulin induces a hyperpolarization and a decreased firing rate of AgRP neurons, thus reducing the release of AgRP and other neurotransmitters, affecting peripheral hepatic innervation, and finally leading to increased interleukin (IL)-6 expression in the liver parenchymal cells (Könner et al., 2007; for a review, see Könner and Brüning, 2012). In the liver, IL-6 action leads to decreased expression of glucose-6-phosphatase and subsequently to reduced gluconeogenesis (Könner et al., 2007).

Adipose tissue-derived leptin, encoded by the *LEP* gene (previously known as *ob*) (Zhang et al., 1994), is released into the plasma in levels proportional to whole-body fat stores (Myers and Olson, 2012) and is induced by insulin (Russell et al., 2001). Leptin plays a crucial role in the central regulation of food intake and energy homeostasis at multiple levels: it directly excites POMC neurons and induces *POMC* expression, while exerting an inhibitory effect on AgRP/NPY neurons and the expression of *AGRP* (see poster, feeding state) (Sohn et al., 2013b). Thus, the net effect of leptin action within the hypothalamus is to inhibit food intake and to increase energy expenditure. Of note, although the actions of insulin and leptin are interconnected at the level of the hypothalamus and both together are required for a complete anorexigenic and glucoregulatory effect, leptin and insulin act on different subpopulations of POMC neurons (Williams et al., 2010), the characterization of which is urgently needed to decipher their precise function (Belgardt and Brüning, 2010; Vogt and Brüning, 2013).

In addition to the ARC, the VMH represents another effector site for leptin and insulin action in the control of energy homeostasis, and recent studies have sought to establish the physiological relevance of these pathways. Mice with insulin receptor deletion in VMH-specific steroidogenic-factor-1 (SF-1) neurons are protected from diet-induced obesity and deterioration of glucose metabolism and, furthermore, display increased POMC neuron activity under high-fat diet conditions (Klöckener et al., 2011). This indicates that high-fat-diet-induced insulin-dependent activation of VMH neurons contributes to obesity development. On the other hand, enhanced leptin receptor signaling in SF-1 neurons within the VMH results in improved glucose homeostasis while body weight is not significantly affected (Zhang et al., 2008). In the DMH, leptin action leads to increased energy expenditure via enhanced sympathetic activation of the BAT, thereby, impacting body weight control independent of food intake (Enriori et al., 2011; Rezai-Zadeh et al., 2014).

Insulin also acts on dopaminergic neurons of the mesolimbic reward system. Here, insulin negatively modulates reward-related behavior such as the desire for high-fat or high-sugar food and reduces hedonic feeding (Könner and Brüning, 2012; Vogt and Brüning, 2013). Likewise, leptin reduces food intake upon injection into the VTA, and ablation of leptin receptors in this area increases the rewarding aspect of highly palatable food (Hommel et al., 2006; for reviews, see Belgardt and Brüning, 2010; Khanh et al., 2014). Intriguingly, a recent study revealed that insulin promotes dopamine release in reward centers in a dynamic fashion. While insulin enhances dopamine release upon food restriction, this effect is lost with an obesogenic diet, indicating that insulin alters food choices depending on the nutritional status of the organism (Stouffer et al., 2015).

Overall, leptin and insulin are major players in the central regulation of energy and glucose homeostasis at various levels within and outside the hypothalamus. Since leptin and insulin receptors are distributed differentially in distinct subsets of central nuclei, a key outstanding challenge is to unequivocally decipher the distinct roles of the different neuronal subsets that express the leptin or insulin receptor, or both together, in terms of central regulation of feeding and glucose control, energy expenditure and reward mechanisms.

### Gastrointestinal hormones

Gastrointestinal hormones have also emerged as important regulators of CNS-dependent energy control. Ghrelin, which is predominantly secreted from the stomach during starvation, robustly stimulates feeding by activating AgRP/NPY neurons, in addition to promoting body weight gain and adiposity via direct effects on PVN neurons (see poster, Healthy fasting state) (Andrews, 2011).

Other hormones, including glucagon-like peptide 1 (GLP-1), peptide YY_3-36_ (PYY_3-36_) and cholecystokinin (CCK), are released from the intestine upon nutrient ingestion and exert anorexigenic effects in various brain regions (such as the ARC, the NTS, the DMH, the VMH and the PVN, see poster, Healthy feeding state) and by modulating vagal afferents, the peripheral components of the gut-brain axis (Sobrino Crespo et al., 2014). However, GLP-1 also acts as a neurotransmitter in the brain (for simplicity, GLP-1 is not depicted separately in the poster). This hormone is produced in distinct neuronal populations within the NTS and centrally influences not only food intake, body weight and glucose homeostasis at the level of the ARC (Sandoval and Sisley, 2015) and the PBN (Richard et al., 2014), but also appears to have a major impact on food reward behavior by directly acting on the VTA and the nucleus accumbens (NAc) (Skibicka, 2013). Thereby, GLP-1 reduces food reward behavior and, if food choice is provided, selectively reduces intake of high-fat chow while increasing low-fat chow intake (Alhadeff et al., 2012). Overall, central GLP-1 receptor activation seems to influence food intake, glucose and energy homeostasis at various levels within the CNS (for reviews, see Burcelin and Gourdy, 2017; Lockie, 2013; Baggio and Drucker, 2014; Katsurada and Yada, 2016). However, more research is needed to unravel the exact molecular mechanisms and cellular effectors of GLP-1 receptor-mediated actions. In light of its multiple beneficial effects on body weight regulation, GLP-1 receptor agonists were very recently approved for the treatment of humans that are obese or overweight (Burcelin and Gourdy, 2017).

## Hypothalamic pathways involved in dysregulated energy homeostasis

### Hypothalamic inflammation, insulin and leptin resistance

Obesity is associated with chronic low-grade inflammation and resistance to leptin and insulin action not only in the periphery but also in the CNS (see poster, Obese feeding state) (Hotamisligil et al., 1993; Könner and Brüning, 2012). Research in murine model organisms has contributed substantially to our present understanding of the hypothalamic mechanisms associated with and contributing to the development of obesity, as outlined below. As an early event in obesity development and even within a few days of ingesting a high-fat, calorie-dense diet, an increased amount of saturated fatty acids (FAs) from the periphery crosses the BBB and induces an inflammatory response in hypothalamic neurons (Thaler et al., 2012). This involves activation of microglia, the tissue-resident macrophages in the CNS. Local inflammation in the mediobasal hypothalamus (encompassing the ARC, the anterior part of the PVN and the ME) promotes endoplasmic reticulum (ER) stress in hypothalamic neurons, leading to insulin and leptin resistance (Pimentel et al., 2014; Kleinridders et al., 2009). It has recently been demonstrated that constitutive activation of the proinflammatory c-Jun N-terminal kinase 1 (JNK1) pathway in AgRP neurons increases spontaneous firing in these cells, along with neuronal and systemic leptin resistance, resulting in hyperphagia (overeating), increased weight gain and adiposity (Tsaousidou et al., 2014). Furthermore, constitutive activation of the inflammatory inhibitor of nuclear factor kappa-B kinase 2 (IKK2) pathway blunts the response of AgRP neurons to insulin and impairs systemic glucose homeostasis (Tsaousidou et al., 2014). Thus, hypothalamic inflammation impairs the effects of insulin and leptin, contributing not only to hyperphagia and obesity development but also to the associated dysregulation of glucose homeostasis.

Although POMC and AgRP neurons in the ARC develop insulin resistance in response to a high-fat diet, SF-1-expressing neurons in the VMH increase their insulin sensitivity, leading to an impairment of the glutamatergic innervation of SF-1 neurons to anorexigenic POMC neurons, resulting in impaired POMC activation. In line with this, ablation of the insulin receptor on SF-1 neurons restores POMC activity and results in a protection from high-fat-diet-induced obesity and the associated deterioration in glucose tolerance (Klöckener et al., 2011; Könner and Brüning, 2012). Similarly, in the obese state, leptin sensitivity is retained in the DMH and increased leptin action on these neurons contributes to obesity-associated hypertension (Simonds et al., 2014) presumably via an increase in sympathetic outflow.

Leptin and insulin resistance in the CNS can also result from overactivation of signal transducer and activator of transcription 3/suppressor of cytokine signaling 3 (STAT3/SOCS3), an intracellular pathway that is activated by leptin (Vaisse et al., 1996). Chronic activation of STAT3, e.g. due to elevated leptin resulting from growing adiposity, leads to increased SOCS3 activation, which in turn inhibits STAT3 signaling in a negative feedback manner resulting in resistance, not only to leptin, but also to insulin (Ernst et al., 2009). Thus, obesity is associated with selective insulin and leptin resistance at the level of the CNS, whereby leptin and insulin action are attenuated in certain neuronal populations while others become more leptin- and insulin-sensitive, thus further promoting hyperphagia, weight gain and a dysregulation in glucose homeostasis (Könner and Brüning, 2012). These findings have given rise to the concept of differential neuronal hormone resistance and hypersensitivity – termed ‘selective hormone resistance’ (Könner and Brüning, 2012).

Collectively, numerous studies in humans have confirmed findings obtained from rodent model organisms that clearly point towards a major role of central insulin resistance in the dysregulation of energy homeostasis and the development of obesity in humans (Heni et al., 2015). However, high-fat-diet-induced obesity is not only associated with a central resistance to insulin and leptin. There is also evidence for the impaired ability of ghrelin to induce food intake at the level of the ARC AgRP/NPY neurons, which could be an adaptive response to prevent further food intake in obese individuals (see poster, Obese fasting state) (Briggs et al., 2010). However, ghrelin-mediated activation of the PVN is retained in obesity (Briggs et al., 2010), which suggests that ghrelin might increase adiposity independent of food intake (Shrestha et al., 2009). Of note, circulating ghrelin levels are lowered in obese humans (Castañeda et al., 2010), presumably again as an adaptive mechanism.

### Maternal obesity – a major risk factor for metabolic deteriorations in the offspring

The number of obese women of reproductive age is increasing worldwide at an alarming rate (Poston et al., 2016). Several observational clinical studies over past decades have revealed a clear association between maternal obesity and the development of metabolic disorders in the offspring (for reviews, see Steculorum et al., 2013; Godfrey et al., 2017). However, the molecular mechanisms that prime offspring of obese mothers for the development of obesity have only recently been investigated in non-human primate and rodent model organisms (Barrett et al., 2016). An elegant study in non-human primates recently demonstrated that high-fat diet intake in obese mothers leads to an overconsumption of high-fat and sucrose-dense (palatable) food, possibly related to an impairment in dopamine signaling and dopaminergic fiber projections to the prefrontal cortex (Rivera et al., 2015). Consistent with this, studies in rat offspring revealed altered food choice towards energy-dense food and associated obesity development (Bayol et al., 2007) as well as alterations in the dopaminergic mesolimbic system and resulting perturbations in reward responses, including an increased response to a fat-enriched reward (Naef et al., 2008, 2011). Strikingly, a maternal diet rich in saturated fatty acids during gestation and lactation leads to hypothalamic inflammation in rodent offspring (Rother et al., 2012; Pimentel et al., 2012). In line with this, maternal high-fat diet intake has also been shown to induce inflammation in the offspring in non-human primates (Grayson et al., 2010).

In rodents, maternal high-fat diet has been shown to impair leptin signaling at the level of the ARC, leading to an attenuated leptin-induced appetite suppression (Kirk et al., 2009). Furthermore, it has been recently discovered that maternal high-fat feeding during lactation in mice severely alters the projections of POMC and AgRP neurons to second-order neurons, resulting in increased obesity and impaired glucose homeostasis in the offspring (Vogt et al., 2014; for review, see Vogt and Brüning, 2013). In the light of the worldwide growing epidemic of overweight and obese children, more research in this field is desperately needed to better understand which maternal-derived factors and underlying mechanisms increase the risk for obesity in the following generation and how these risk factors can be tackled.

### Mitochondrial function and dynamics in obesity

Mitochondria have emerged as important players in the development of a number of metabolic disorders including obesity, type 2 diabetes mellitus, and cardiovascular diseases (Wai and Langer, 2016). Mitochondria regulate cellular energy and fuel metabolism in a highly dynamic fashion, enabling fast adaptation to changes in cellular energy supply and demand. In the hypothalamus, mitochondrial reactive oxygen species (ROS) have emerged as important signaling molecules that indicate positive or negative energy states at the level of AgRP/NPY and POMC neurons (see poster) (Shadel and Horvath, 2015). In the negative energy or fasting state, when systemic glucose levels are low, the activation of AgRP/NPY by ghrelin leads to an induction of long-chain fatty acid β-oxidation and concomitant ROS production in AgRP/NPY neurons, which activates uncoupling protein (UCP)2 expression (Andrews et al., 2008). UCP2, in turn, reduces intracellular ROS levels, enabling a sustained energy supply from β-oxidation without ROS generation and thereby promoting sustained AgRP/NPY firing, which results in increased food intake (Andrews et al., 2008). In the positive postprandial energy state, when systemic glucose levels are high, glucose is taken up and oxidized by POMC neurons, leading to the generation of ATP and ROS, and, in turn, to depolarization and increased firing of these neurons, promoting satiety and a decrease in food intake (Diano et al., 2011). Fluctuating hypothalamic ROS levels not only direct hunger and satiety and ultimately food intake, but also energy expenditure and peripheral glucose utilization (Shadel and Horvath, 2015). Upon exposure to a high-fat diet, ROS levels plateau, resulting in impaired satiety signaling in POMC neurons (see poster, Obese feeding state) (Shadel and Horvath, 2015). Mitochondria also constantly adapt to changes in cellular energy levels and fuel availability via conformational changes, so-called ‘fission and fusion’ events and via interaction with the ER (Nasrallah and Horvath, 2014). Mitofusins (MFNs), highly conserved mitochondrial transmembrane GTPases, are of central importance for the regulation of these processes (Schrepfer and Scorrano, 2016). There are two mammalian mitofusins, MFN1 and MFN2. Both have been implicated in mitochondrial fusion processes, while MFN2 also specifically controls ER morphology and its interaction with mitochondria (Schrepfer and Scorrano, 2016; Wai and Langer, 2016). One study has shown that deletion of MFN1 or MNF2 in AgRP/NPY neurons protects mice from high-fat diet-induced obesity (Dietrich et al., 2013). Deletion of MFN2 in POMC neurons resulted in deprived mitochondria-ER contacts, leptin-resistance, hyperphagia, reduced energy expenditure and morbid obesity (Schneeberger et al., 2013). Interestingly, POMC-specific knockout of MFN1 did not result in any alteration of energy, or glucose homeostasis (Schneeberger et al., 2013). This striking difference in POMC-specific MFN2 versus MNF1 deletion on the metabolic outcome phenotype might indicate the importance of ER integrity and coordinated ER-mitochondrial interactions for proper POMC function.

Taken together, these findings demonstrate the crucial role of mitochondrial function and dynamics not only in the regulation of basal biodynamic processes such as satiety, hunger and whole-body metabolism but also in disease development, suggesting that mitochondrial dynamics could represent novel therapeutic targets in obesity treatment. In support of this, berberine, a natural plant product, used in Chinese medicine, inhibits mitochondrial respiration (Turner et al., 2008; Xu et al., 2014) and has been shown to effectively improve energy and glucose metabolism in insulin-resistant, obese rodents (Lee et al., 2006) and humans (Wei et al., 2012). In addition, Metformin, a first-line treatment in type 2 diabetes (American Diabetes Association, 2016) with neutral effects on body weight, is supposed to exert its antidiabetic effects, at least in part, via inhibition of mitochondrial respiration (Owen et al., 2000; El-Mir et al., 2000). Consistent with this, direct application of metformin to the brain in rodents has been shown to decrease food intake and improve glucose metabolism (Portela et al., 2015).

### Hypothalamic gene expression in obesity

In accordance with their role in anorexic signaling pathways, mutations in the *MC4R* and *POMC* genes, which encode the melanocortin 4 receptor and pro-opiomelanocortin, respectively, result in hyperphagia and obesity in humans (Krude et al., 1998; Vaisse et al., 1998; Yeo et al., 1998) and rodents (Challis et al., 2004; Huszar et al., 1997; Yaswen et al., 1999; Yeo and Heisler, 2012). The most severe form of obesity in mice and humans results from a deficiency in the genes encoding leptin (*LEP*) or the leptin-receptor (*LEPR*) (Friedman and Halaas, 1998; Zhang et al., 1994; Chen et al., 1996; Montague et al., 1997). Importantly, leptin-deficiency-associated obesity successfully resolves upon treatment with recombinant leptin (Farooqi et al., 1999; Pelleymounter et al., 1995), clearly indicating the major physiological role of leptin in the regulation of whole-body energy homeostasis. Remarkably, mutations underlying monogenetic obesity in humans have been identified not only in the *POMC/MC4R* genes, but also in transcription factors and neurotransmitters that modulate the activity of the homeostatic melanocortin circuitry, such as single-minded homolog 1 (*SIM1*) (Bonnefond et al., 2013; Ramachandrappa et al., 2013), brain-derived neurotrophic factor (*BDNF*) (Gray et al., 2006; Han et al., 2008, Xu et al., 2003) and tyrosine receptor kinase B (encoded by *NTRK2*) (Yeo et al., 2004), highlighting their important role in the physiological regulation of energy homeostasis.

Interestingly, genome-wide association studies in humans have revealed a robust association of single nucleotide polymorphisms (SNPs) with excess weight and obesity, not only in or near to the *BDNF* gene (for a review, see Xu and Xie, 2016) but also in intron 1 of the fat mass and obesity-associated protein (*FTO*) gene (Dina et al., 2007; Scuteri et al., 2007; Frayling et al., 2007; for a review, see Hess and Brüning, 2014). It was recently shown that deletion of *Fto* in rodents impairs dopaminergic control of behavioral and neural responses and alters methylation of several neural mRNAs, leading to an altered protein expression level of the encoded proteins and an overall changed dopaminergic midbrain circuitry response, pointing to a role of the *FTO* gene in complex behaviors like reward-based decision-making (Hess et al., 2013). The notion that *FTO* controls dopaminergic signaling has since been confirmed in human fMRI studies (Sevgi et al., 2015). Overall, ongoing and future research exploring genetic variants in obese humans will allow the identification of novel candidate genes leading to the discovery of potential new drug targets for the treatment of obesity.

## New perspectives in CNS-dependent control of feeding behavior and metabolism

A recent report in rodents demonstrated that AgRP neurons are activated by uridine-diphosphate (UDP), which is synthesized from circulating uridine in the CNS by the nucleotide salvage pathway (Steculorum et al., 2015). UDP acts on AgRP neurons via the purinergic receptor 6 (P2Y6) to promote feeding (Steculorum et al., 2015). In the context of obesity, uridine levels are elevated in the circulation (Steculorum et al., 2015), indicating that the uridine/UDP system might be another mechanism that promotes the vicious cycle of increased weight gain along with exaggerated food intake. Consistent with this model, AgRP-neuron-specific P2Y6-deficient mice are protected from high-fat-diet-induced adiposity and insulin resistance (Steculorum et al., 2017) (see poster, Obese fasting state).

A recent study conducted in mice challenged the idea that POMC neurons are solely anorexigenic by showing that activation of the cannabinoid receptor 1 on POMC neurons leads to the release of β-endorphin, an opioid neuropeptide, which in turn promotes feeding (Koch et al., 2015). This anorexigenic-to-orexigenic switch of POMC neurons might be responsible for cannabinoid-induced hyperphagia (Waterson and Horvath, 2015).

Another concept that has recently come to light via studies of rodent model organisms is the active role of astrocytes (glial cells in the nervous system) in the regulation of feeding and glucose homeostasis. First, it was demonstrated that astrocytes are involved in leptin-dependent control of feeding, as ablation of leptin-receptor signaling in astrocytes blunted the anorexic effect of leptin and enhanced fasting and ghrelin-induced hyperphagia (Kim et al., 2014). Second, insulin-receptor signaling in astrocytes was demonstrated to play a crucial role in glucose uptake into the brain, glucose sensing in the hypothalamus, and feeding behavior, as astrocyte-specific deletion of the insulin receptor resulted in increased food intake and deteriorated glucose tolerance (García-Cáceres et al., 2016). In addition, it was recently shown in mice that activation of medial hypothalamic astrocytes directly leads to the inhibition of ghrelin-independent as well as ghrelin-induced food intake via the inhibition of AgRP/NPY neurons (Yang et al., 2015). The study also revealed that inhibition of AgRP/NPY neurons by astrocytes is mediated via increased extracellular levels of adenosine (derived from ATP that is released by astrocytes upon activation), which acts on the adenosine A1 receptor on synaptic AgRP/NPY endings, leading to silencing of AgRP/NPY neurons.

Collectively, these new perspectives on novel regulatory mechanisms in the central control of energy and glucose metabolism highlight that we are only starting to understand how whole-body energy homeostasis is regulated and which neuronal and non-neuronal components are involved.

## Therapeutic avenues for obesity

The picture that researchers are gradually building of the CNS mechanisms underlying dysregulation of energy and glucose homeostasis is contributing to the development of new candidate therapies for obesity and related disorders. A very promising therapeutic strategy is emerging from pre-clinical and clinical studies with proglucagon-derived dual or triple co-agonists (for an overview of clinical and preclinical studies, see Tschop et al., 2016). These poly-agonists, consisting of peptide hybrids of a GLP-1 receptor (GLP-1R) agonist and a glucagon receptor (GcgR) agonist (GcgR/GLP-1R) or a GLP-1R agonist together with an agonist for gastric inhibitory polypeptide (GIP) receptor, another important metabolic hormone in glucose homeostasis (GIPR/GLP-1R), exhibit higher efficacy in terms of body weight reduction and glycemic control while giving rise to fewer side effects compared with equimolar doses of mono-agonists in rodents and humans (Finan et al., 2013). Furthermore, preclinical studies with a GcgR/GIPR/GLP-1R triple agonist revealed even more promising results in terms of body weight and glucose control (Finan et al., 2015).

Another encouraging therapeutic approach is the use of a selective MC4R agonist, which has been proven successful in the treatment of *POMC*-deficient obesity (Kuhnen et al., 2016) as well as in humans with non-genetic obesity (Chen et al., 2015a) and in non-human primates (Kievit et al., 2013). However, Phase 2 and Phase 3 studies with a sufficient number of patients enrolled are needed to elucidate if these therapeutic approaches show real promise in terms of effectiveness and side effect profile.

## Conclusions

To tackle the growing obesity epidemic and its associated metabolic disorders, there is an urgent need to further decipher the CNS-level molecular mechanisms that lead to the dysregulation in energy homeostasis. This could set the stage for the development of new therapeutic strategies. One of the most intriguing mysteries that needs to be unraveled is how maternal metabolism impacts neurodevelopmental aspects of the fetus that predispose to obesity and metabolic dysfunction later in life. Moreover, it is becoming increasingly apparent that neuronal populations are not uniform but consist of clustered neurons with very different, unique entities that often result in opposing properties; thus, it is of high importance to fully characterize these neuronal sub-populations and to unravel their distinct features and projections to allow for specific therapeutic targeting. Another high priority question is how the well-known neuronal centers involved in feeding control, such as the hypothalamic nuclei, are regulated by and connected to superordinate brain centers including the reward system and sensory organs (e.g. the gustatory and olfactory systems). It is also important to unravel the role of non-neuronal, CNS-resident cell entities in the regulation of feeding and central metabolism. In summary, it is apparent that dysregulation of energy metabolism in the context of disease is far from being a single-track process allowing for easy and simple solution approaches. Therefore, a broadened view, integrating the different aspects of obesity development is a prerequisite to successfully fight obesity in the future.
